# Apnea of Prematurity and Oxidative Stress: Potential Implications

**DOI:** 10.3390/antiox13111304

**Published:** 2024-10-27

**Authors:** Lauren Thompson, Joseph W. Werthammer, David Gozal

**Affiliations:** Department of Pediatrics, Joan C. Edwards School of Medicine, Marshall University, Huntington, WV 25701, USA; herman8@marshall.edu (L.T.); werthammer@marshall.edu (J.W.W.)

**Keywords:** apnea of prematurity, intermittent hypoxia, CPAP, oxidative stress, retinopathy of prematurity, bronchopulmonary dysplasia, necrotizing enterocolitis, cognitive development, supplemental oxygen

## Abstract

Apnea of prematurity (AOP) occurs in 85% of neonates ≤34 weeks of gestational age. AOP is frequently associated with intermittent hypoxia (IH). This narrative review reports on the putative relationship of AOP with IH and the resulting oxidative stress (OS). Preterm infants are susceptible to OS due to an imbalance between oxidant and antioxidant systems with the excessive free radical load leading to serious morbidities that may include retinopathy of prematurity, bronchopulmonary dysplasia, and neurodevelopmental delay. Current therapeutic approaches to minimize the adverse effects of AOP and optimize oxygen delivery include noninvasive ventilation and xanthine inhibitor therapy, but these approaches have only been partially successful in decreasing the incidence of AOP and associated morbidities.

## 1. Introduction

Preterm birth, defined as delivery before 37 weeks gestation, is a global public health issue of significant magnitude. In 2020, an estimated 13.4 million neonates were born prematurely, accounting for more than 10% of births worldwide [1]. Advances in medical care have led to improved survival at increasingly earlier gestations, but this technological success has come with a rise in the incidence of chronic morbidities among the surviving high-risk neonates.

Common morbidities associated with premature birth include chronic lung disease, retinopathy of prematurity (ROP), sleep-disordered breathing, neurodevelopmental delay, growth failure, necrotizing enterocolitis (NEC), and cardiovascular dysfunction. Recent emerging evidence has pointed to oxidative stress (OS) as a major contributing factor underlying these morbidities, which occurs when there is an imbalance between oxidant and antioxidant systems [2]. Preterm infants are particularly susceptible to OS due to the underlying scarcity in the expression and activity of antioxidant enzymes and consequently immature antioxidant responses. In addition, preterm infants are also exposed to environmental factors that foster a pro-oxidant state such as the need for oxygen supplementation and parenteral nutrition [3]. Clinically, apnea of prematurity (AOP) and the resultant intermittent hypoxia (IH) that characterizes this condition will potentiate the OS that occurs in preterm neonates and if allowed to persist for extended periods, may promote the risk of multisystem complications.

In this review, we will discuss (1) the magnitude of the problem of AOP and IH, (2) the relationship between IH and OS, (3) the currently identified associations between OS and common morbidities seen in the preterm neonate, and (4) the relatively scarce therapeutic approaches to mitigate IH and OS [4].

## 2. Apnea of Prematurity—The Magnitude of the Problem

**Figure 1:**
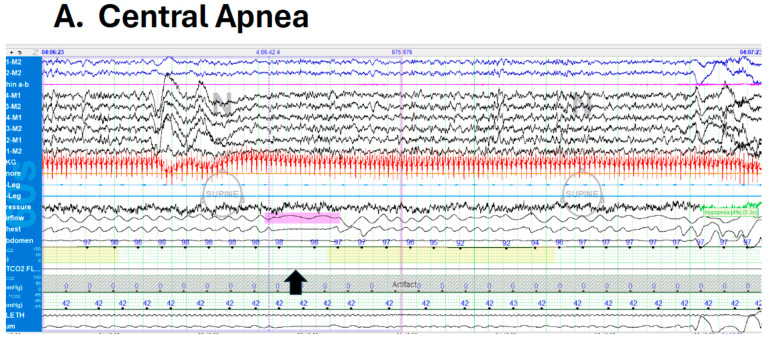

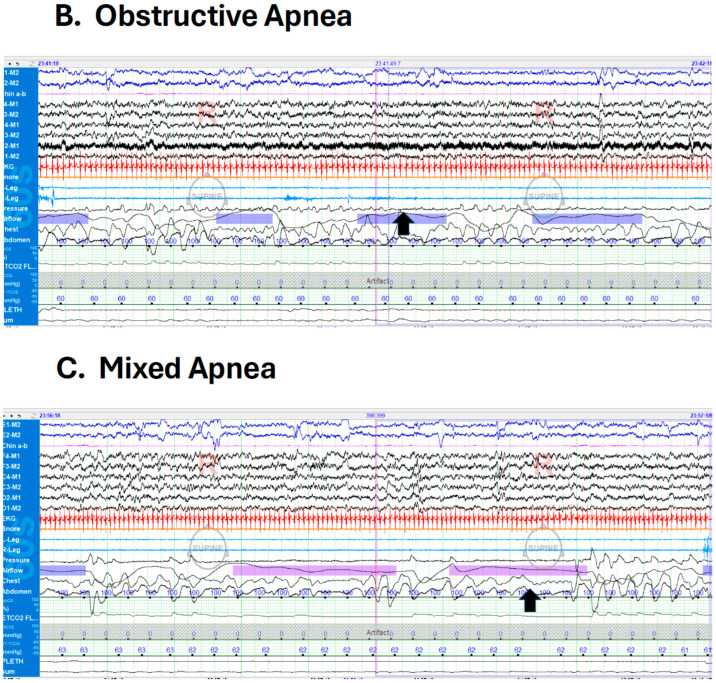
Examples representing 3 types of apneas of prematurity events. Arrows indicate apneas, and colored highlights in pink, purple, and magenta indicate respective events. (**A**)—central apnea: 10 s central apnea with absence of both airflow by nasal thermistor and chest wall movement by transthoracic impedance followed by drop in SpO2 to 92%. (**B**)—obstructive apnea: 9 s obstructive apnea with absent airflow with cardiac deceleration with continued chest wall movement. (**C**)—mixed apnea: 20 s mixed apnea with initial obstruction with absent airflow with chest wall movement followed by central apnea with cardiac deceleration.

Apnea of prematurity (AOP) is one of the most common pathologies seen in premature infants affecting 85% of neonates born ≤34 weeks gestation [5]. The prevalence of AOP is inversely proportional to gestational age, with virtually all infants born ≤28 weeks being affected compared to 20% of the infants born at 34 weeks gestation [6]. AOP generally resolves spontaneously around 36–43 weeks of gestational age, although the age at which AOP resolution will occur is stringently dependent on the degree of prematurity at birth, such that very extreme premature infants may manifest AOP well into 50 weeks post-conceptional age [6,7,8]. Clinically significant AOP has been defined as breathing pauses that last >20 s or for >10 s if associated with bradycardia (e.g., <80 beats per minute) or oxyhemoglobin desaturation (e.g., O_2_ saturation <80–85%) [9]. AOP, i.e., the cessation of airflow, has traditionally been classified as (a) central, characteristically manifesting as the absence of respiratory efforts, (b) obstructive, as illustrated by the presence of ongoing inspiratory efforts in the context of upper airway obstruction, and (c) mixed, whereby a combination of central apnea is either preceded or followed by upper airway obstruction. (Figure 1). Among the obstructive types of AOP, the site of obstruction in the upper airways is primarily in the pharynx, although it may also occur at the larynx and possibly at both sites [10]. All three subtypes of AOP are related to some degree of brainstem immaturity along with contributions and interactions with respiratory mechanics [11,12,13,14,15,16,17]. Mixed apnea events with loss of upper airway patency following a central pause are the most common type in premature infants [18,19].

The pathogenesis of AOP is multifactorial and not fully understood [20,21,22]. AOP may simply resemble the fetal breathing pattern, where breathing is irregular because its main function is to ensure lung growth rather than promote gas exchange [22]. Central nervous system mechanisms and peripheral reflex pathways have all been implicated in the pathophysiology of AOP [8,10,23] (Table 1).

Reduced central chemoreceptor responsiveness and instability of the central respiratory pattern generator along with enhanced peripheral chemoreceptor function have all been proposed as contributors to the initiation of apnea events and also to its continuation until some intervention is implemented [10,21]. The dysmaturity of the brainstem respiratory pattern generator and altered ventilatory responses to hypercarbia and hypoxia are all likely to contribute to AOP [24,25,26]. Changes in CO_2_ are sensed at many different central nervous system regions even though the initial discoveries pointed to three different sites at or near the ventral medullary surface of the brainstem; the current consensus suggests that there is a multiplicity of chemosensitive fields collectively contributing to the integrative cardiorespiratory responses along with the preservation of respiratory drive [27,28,29,30,31,32,33,34,35]. Preterm infants have long been recognized to exhibit a diminished response to CO_2_ when compared to more mature infants [14]. Furthermore, the carotid bodies and their developmentally regulated maturational patterning are critically important to the sensing of changes in oxygen levels as well as serving as the major peripheral chemical driver of respiration at all ages [10,36,37]. Peripheral chemoreceptors undergo a very coordinated developmental trajectory initiated by the transition from the hypoxic fetal environment to the environmental air-oxygenated milieu, a phenomenon commonly referred to as “resetting” [38,39]. In settings such as preterm birth in which ventilatory support is required and supplemental oxygen is administered, the resetting phenomenon may be affected and such blunting of peripheral chemoreceptor responses may be long-lasting and involve prolongation of apneic events [40,41,42].

As mentioned above, peripheral chemoreceptors primarily in the carotid body are responsible for stimulating breathing in response to hypoxia. The classic hypoxic response in the preterm neonate is triphasic, consisting of an initial increase followed by a decrease in ventilation usually ascribed to centrally mediated inhibitory inputs [14,43]. However, preterm infants <1500 g show a virtually absent stimulatory response and instead manifest an immediate reduction in minute ventilation during hypoxia, which is mainly secondary to a decrease in respiratory rate related to central nervous system inhibition [44].

Apnea of prematurity and the resulting intermittent hypoxia (IH) it causes are exceedingly common in the preterm neonate and have been recognized as such with the advent of technology capable of continuously measuring oxygen saturation levels. Historically, invasive arterial blood gas sampling was the gold standard for measuring oxygen levels in the NICU. However, these sporadic estimates cannot identify the frequent fluctuations in oxygen levels that occur in preterm infants. In contrast, transcutaneous and pulse oximetry monitoring can provide continuous monitoring of an infant’s oxygen levels. Transcutaneous monitoring requires periodic calibration and involves heating the membrane positioned on the skin of the infant at the probe site, leading pulse oximetry to become adopted as the standard practice for cardiorespiratory monitoring in the neonatal intensive care unit (NICU). However, pulse oximetry is not without flaws and has limited value in identifying periods of hyperoxemia with the use of supplemental oxygen. Pulse oximeters also have decreased accuracy at lower levels of SpO2. Furthermore, the averaging time used by the embedded algorithm in the instrument can affect accurate characterization of apneic and hypoxic events. While a short averaging time produces the most accurate waveform, a longer averaging time is used in many NICUs to reduce the high frequency of alarms. This can, for example, portray a hypoxic event as an extended event, when in fact it consists of multiple short, self-resolving events occurring in a cluster. This is concerning for two reasons. First, a longer event is more likely to receive supplemental oxygen that may be unnecessary. Second, there is increasing evidence that clustered IH events versus dispersed events are likely associated with different clinical outcomes. Given these challenges, it is difficult to accurately measure and record the true burden of these various types of events and develop informed predictions as to their consequences [3].

Martin et al. [45] tackled this challenge by following a cohort of 79 preterm infants (24–28 weeks gestation) over the first 8 weeks of postnatal life using continuous high-resolution pulse oximetry data (2 s sampling rate and 2 s averaging time). They identified desaturation events ≤80% from between 10 s and 3 min and calculated the number of events for each infant for each week of postnatal age. Their results displayed the presence of an abrupt increase in the density of events after the first week of life followed by a progressive increase in weeks 2–4 reaching ~600 events/week and finally a decrease by weeks 6 to 8 of postnatal life. The authors hypothesized that this trend is due to the decline in central chemosensitivity during the first week of postnatal life. They further concluded that the high incidence of IH episodes in this population (50–100 per day) would likely be underestimated using conventional pulse oximetry settings [45]. Thus, IH is an extremely frequent condition in premature infants and can be the consequence of initially delayed maturation and resetting of the peripheral chemoreceptors followed by rebound hypersensitivity of these receptors [34,36].

## 3. Oxidative Stress Cellular Mechanisms

Oxidative stress is usually considered as the presence of “a serious imbalance between the generation of reactive oxygen species (ROS) and antioxidant defenses in favor of ROS, causing excessive oxidative damage to biomolecules and cellular organelles”. In a biological cellular context, ROS are generated as a natural by-product of aerobic metabolic pathways. Accordingly, mitochondrial respiration is a substantial source of ROS [46]. In addition to mitochondria, ROS are synthesized by a variety of enzymes such as the NADPH oxidase family, xanthine oxidase, nitric oxide synthase, and peroxisomal constituents [47]. In addition, ROS are generated during exposure to ionizing and UV radiations, as well as by the ingestion and activity of a wide range of drugs and xenobiotics. In the endoplasmic reticulum, oxidative moieties are released during the folding of proteins and the formation of disulphide bonds. ROS are characteristically very reactive chemical molecules particularly in their ability to aggregate electrons to the oxygen molecule [48]. In this context, unstable molecules such as superoxide anion (•O_2_^−^), hydrogen peroxide (H_2_O_2_), hydroxyl radical (OH^−^), and singlet oxygen (^1^O_2_^−^) are produced. We should emphasize that ROS are intrinsic to the homeostatic functioning of cells, fulfilling critically important functions [49]. However, when ROS exceed antioxidant capacity, oxidative stress (OS) damage occurs [50].

## 4. Relationship Between IH and OS

As discussed above, oxidative stress (OS) occurs because of the homeostatic imbalance between ROS production and the efficacy of antioxidant systems in canceling OS, thereby leading to an increased and excessive level of free radicals with subsequent damage to cells and organs [2,51]. A free radical is any atom or molecule that contains one or more unpaired electrons that includes reactive oxygen species (ROS), reactive nitrogen species (RNS), and sulfur-centered radicals collectively named oxidants [2,52]. These molecules are highly unstable, highly reactive, and known to cause direct damage to a variety of cellular structures. Under normal physiological circumstances, only a small percentage of oxygen is incompletely reduced to form ROS, and most ROS are neutralized by endogenous or exogenous antioxidants. At low physiological levels, free radicals can be protective and, for example, play an important role in our immune system. However, increased levels of free radicals are produced in response to stressful metabolic situations and can cause oxidative and nitrosative stresses that directly damage tissues [3]. Infections, hypoxia–reoxygenation, ischemia, inflammation, hyperoxia, and radiation are stressors commonly known to induce oxidative stress. In addition to the direct cellular damage caused by ROS and RNS, they have also been shown to trigger pro-inflammatory and pro-apoptotic pathways that accentuate and propagate the damage [3,53,54].

There are several reasons why newborn infants, especially those born preterm, are at risk of oxidative stress. First, relative oxidative stress is generally high within the first weeks of birth after transitioning from the low-oxygen fetal environment of the uterus (PaO2 20–26 mm Hg) to the rich extrauterine oxygen environment (PaO2 100 mm Hg) [55,56]. Second, preterm infants have insufficient antioxidant defenses either de novo or passively acquired transplacentally until the third trimester, which increases their susceptibility to oxidative stress [56,57]. Third, premature infants have a high energy demand for growth and very high metabolic rates, which in the process of generating ATP (adenosine triphosphate) will lead to oxidative product formation in the mitochondria. Lastly, they have a high concentration of non-protein-bound iron (NPBI) that is available to react with H_2_O_2_ through the Fenton reaction and produce hydroxyl radicals [2].

Intermittent hypoxia (IH) is one of the most important consequences of AOP and the culprit for oxidative stress [58]. Indeed, it is now well established that a balanced oxygen homeostasis is an essential requirement to maintain the regulation of normal cellular systems. Any alterations in the oxygen levels are accompanied by cellular stress responses which may modify the expression of a large number of regulatory genes and proteins. If hypoxia occurs, i.e., critical oxygen delivery to preserve cellular bioenergetics, this will result in oxidative stress and the formation of hypoxia-inducible factors (HIFs) and reactive oxygen species (ROS) [59]. The cumulative evidence indicating that IH leads to an overall pro-oxidant state is derived from animal models that illustrate that IH increases extracellular superoxide concentration, activates NADPH oxidases, induces hypoxia-inducible factor 1 (HIF-1α), and downregulates both HIF-2α and superoxide dismutase [60,61,62]. Furthermore, it is known that during hypoxia, ATP, the major cellular source of energy, is depleted. This leads to the formation and accumulation of purine derivatives and hypoxanthine. Upon reoxygenation, oxidases are activated, especially the NADPH oxidase family and xanthine oxidases. These oxidases metabolize purine derivatives and generate a burst of superoxide anion, causing significant oxidative stress and subsequent tissue damage [3]. Beyond the direct action of free radicals on cellular components, oxidative stress has also been shown to cause direct alterations of DNA through the interruption of classical gene expression and DNA regulation of DNA repair, in addition to epigenetic modifications [2]. However, the impact of cycle duration, the severity of oxygen deprivation for each cycle, pattern of re-oxygenation, and overall cumulative duration of the intermittent events will need to be explored in much greater detail to deduce a more specific in-depth understanding of how these elements individually and collectively contribute to OS.

The extent of oxidative stress is difficult to quantify in preterm infants because free radicals are highly reactive and unstable, making them difficult to measure directly. Fortunately, the levels of by-products from the oxidation of lipids, proteins, and DNA can be quantified and have shown promising clinical value [63]. In addition, other biomarkers have been identified that help measure the risk of OS. Those currently applicable to neonatal medicine are summarized in Table 1.

## 5. Clinical Implications of AOP and OS

### 5.1. Retinopathy of Prematurity (ROP)

The largest body of evidence implicating OS in neonatal disease revolves around retinopathy of prematurity (ROP). ROP is a vasoproliferative retinal disorder affecting premature infants and is a leading cause of visual impairment and blindness worldwide. Despite robust screening protocols and treatment advancements, at least 50,000 children worldwide suffer from blindness annually due to this condition [64]. Gestational age and birth weight are the two risk factors most highly correlated with ROP, both being inversely proportional to ROP incidence. Hyperoxia has long been accepted as the primary contributor to the pathogenesis of ROP through increased ROS and RNS and the subsequent vascular damage and excessive angiogenic effect they cause. More recently, studies in both animal models and premature infants have demonstrated that intermittent hypoxia is also a major contributing factor by further increasing oxidative stress in the preterm retina [65].

Upon birth, the neonate must transition from a relatively hypoxic intrauterine environment to an oxygen-rich extrauterine environment. This transition has important consequences for the preterm retina, whose development is not yet complete. In utero, retinal angiogenesis begins around the 15th week of gestation, originating from the optic nerve and expanding peripherally. The nasal portion of the retina does not become vascularized until 36 weeks of gestation and the temporal area not until 40 weeks gestation [64]. Thus, the preterm retina must develop in a maladaptive hyperoxic environment for which it was not programmed. Further occurrence of hypoxic–hyperoxic events, as is the common corollary of AOP, would further challenge the normal maturation of the retina and promote oxidative stress damage translating into the emergence of ROP as demonstrated by several experiments in animal models and confirmed by clinical cohort studies [3,66,67,68,69].

The development of ROP is frequently described as a two-phase occurrence. The first phase is characterized by cessation of normal retinal vascular development due to the hyperoxic extrauterine environment. This occurs largely due to suppression of hypoxia-inducible factor 1α (HIF1-α), which causes downregulation of the vascular endothelial growth factor (VEGF). Oxidative damage to the existing vasculature has been implicated during this stage. The second phase is comprised of abnormal vascular proliferation stimulated in part by the hypoxia-induced production of VEGF and other growth factors [45]. It is most likely during this second stage that IH contributes to ROP by precipitating retinal neovascularization.

Historically, knowledge surrounding hyperoxia and its deleterious effects on the developing retina was the main focus of ROP prevention. Significant debate exists regarding the ideal target oxygen saturation required to mitigate this disease without worsening other outcomes. Studies have found that preterm infants with a lower target oxyhemoglobin saturation (85–89%) compared to a higher target SpO2 (91–95%) have a lower incidence of ROP but incur higher mortality [70]. Thus, other improved options for prevention are needed.

Recent evidence of the impact of IH on developing ROP provides a potential target for prevention. As mentioned, IH creates a pro-oxidant environment through increases in superoxide concentrations and HIF-1α expression, as well as degradation of HIF-2α and downregulation of superoxide dismutase [71]. In preterm infants, Di Fiori et al. demonstrated an association between chronic IH and severe ROP requiring laser therapy [64]. Similarly, a post hoc analysis of the Canadian Oxygen Trial showed that infants experiencing greater frequency or severity of IH events incurred a higher risk for developing ROP [72]. Furthermore, research regarding the timing and patterns of IH have demonstrated that these may be critical in predicting ROP risk. For example, rats exposed to cycles of IH in a clustered manner (three episodes 10 min apart) vs. equally dispersed cycles (2 h apart) will develop more severe retinopathy [73]. These observations provide valuable insights to guide further research regarding preventative strategies and risk stratification for preterm infants at risk for ROP.

### 5.2. Neurodevelopmental Impairment (NDI)

The brain is one of the most sensitive organs to oxidative damage due to the large amount of polyunsaturated fatty acids, the high metabolic rate and bioenergetic dependence on utilization of oxygen, and the relative scarcity of antioxidant defense mechanisms [74]. Mitochondria and NADPH oxidase are the predominant sources of ROS in the central nervous system and have been shown to play a prominent role in IH-induced neuronal injury [74].

The preterm brain is at particularly high risk given the critical period of rapid brain development that occurs postnatally. It is known that oligodendrocyte maturation, myelination, axon development, and synapse formation occur mainly during the third trimester of gestation and in the first postnatal year and thus oxidative stress occurring during this time likely contributes to the white matter impairments frequently seen in preterm neonates [75].

Increased frequency of IH has been shown to be associated with neurodevelopmental impairment, such as language, cognitive, and motor delays. This has been demonstrated in many single-center studies, as well as in the large, multicenter Canadian Oxygen Trial that examined a cohort of >1000 infants [71]. However, data from rodent studies suggest that this relationship is more than just an association. Several studies have shown that in neonatal rodents, IH exposures evoke disruption in sleep architecture and cause working memory impairments and hyperactivity behaviors, remarkably resembling attention deficit hyperactivity disorder (ADHD), a condition frequently manifested among children who were born prematurely [76,77,78,79]. These behaviors were accompanied by a reduction in extracellular levels of dopamine, increased cerebral expression of caspase-3 reflecting increased apoptosis, and marked increases in oxidative stress, neuronal cell losses, and glial proliferation in the CA1 hippocampal region and cortex that could be mitigated by transgenic ablation of NADPH oxidase and inducible nitric oxide synthase, two well-established enzymes that underlie the major generation of ROS [76,80,81]. Subsequent studies led by Gozal and collaborators led to the development of a neonatal mouse model of IH simulating AOP and demonstrated that IH during postnatal days 2–10 induced hypomyelination in the corpus collosum, striatum, fornix, and cerebellum, as well as alterations in myelin-forming processes [76]. A later study by Darnall et al. also reported decreases in white matter integrity in rat pups exposed to IH similar to that experienced by a premature neonate with AOP [82]. Furthermore, preterm infants have recently demonstrated a significant decrease in EEG (electroencephalogram) amplitude during breathing pauses from 5 to >15 s. It was speculated that frequent disruption to brain activity may impact long-term neurodevelopment [83]. An excellent summary of extant studies on the association between IH and nervous system morbidity further confirms the putative adverse effects of IH in prematurely born infants [84].

There are several biomarkers linking oxidative stress to CNS (central nervous system) dysfunction and to white matter injury as seen in preterm neonates [85]. Additionally, non-protein-bound iron (NPBI) has been described as a reliable early predictive biomarker of neurodevelopmental outcomes [86]. Increased levels of isoprostanes (IsoPs) have been reported in preterm infants who developed white matter injury seen at corrected term gestational age. Increased levels of IsoPs have also been found to be associated with poor neurodevelopmental trajectories at 12 months of age [87]. Lastly, augmented adenosine (Ado) plasma level concentration at day 15 in premature neonates has also been found to be significantly associated with white matter injury using MRI. Ado represents a promoter of oligodendrocyte maturation and has previously been reported to be elevated in response to OS in preterm neonates [2].

### 5.3. Respiratory Disorders

Premature neonates, particularly those born less than 28 weeks, have significant short- and long-term respiratory pathology including respiratory distress syndrome (RDS) and increased risk for developing bronchopulmonary dysplasia (BPD). Infants born prematurely have a limited surface area for gas exchange as well as a limited surfactant which is necessary to keep the lungs from collapsing during exhalation. Thus, premature neonates frequently require positive pressure respiratory support, both invasive and noninvasive, and supplemental oxygen. These therapies can increase the risk for future respiratory pathology, making it challenging to distinguish the impact of IH on such pathologies. However, evidence exists that IH plays a role in both short- and long-term respiratory morbidity. Furthermore, significant lung development and alveolarization occurs postnatally for these infants albeit with curtailed pulmonary vascular angiogenesis [88,89], making the lungs a prime target for oxidative stress during the early postnatal period, similarly to other rapidly developing organ systems [90,91,92,93].

Several studies have demonstrated a relationship between the frequency and duration of IH in very-low-birthweight infants and BPD. Animal models have indicated that IH alone is unlikely to cause the parenchymal changes seen in BPD, but that it likely causes a more severe phenotype [94,95,96]. Wheezing and asthma-like symptoms are common sequelae for preterm neonates, affecting over 30% [3]. Di Fiori et al. found that early exposure to IH during the first week of life was associated with reported asthma medication use at 2 years of age [97].

Biomarkers of OS have been found in infants with these diseases, further providing evidence on the putative role of IH and subsequent OS in their pathology [98,99]. In preterm infants with RDS, elevated plasma levels of MDAs (methyl-D-aspartate), protein carbonyls, AOPPs, 8-OHdG, and H_2_O_2_ and an elevated oxidant/antioxidant ratio (calculated as protein carbonyls/(superoxide dismutase + glutathione peroxidase)) were found compared to healthy preterm controls [2]. Another study reported that elevated AOPPs (advanced oxidation protein products) and 8-OHdg on days 0–3 correlated with severity of RDS. For preterm infants who developed BPD, higher urinary levels of 8-OHdg were reported compared to those who did not go on to develop BPD. A higher concentration of 8-OHdg has also been reported in serum and tracheal aspiration samples on the day after birth and at day 28 of life in those infants who developed BPD compared to those who did not [2].

In addition to BPD, prematurity has also been implicated as a risk factor for sleep-disordered breathing later in life [100]. The mechanisms underlying this association are unclear, but it is very possible that bone growth patterns may be affected by IH and lead to maladaptive development of the oropharyngeal space and of the craniofacial structures [101,102,103,104,105,106,107,108,109].

### 5.4. Necrotizing Enterocolitis (NEC)

NEC is a gastrointestinal disorder that primarily occurs in preterm neonates and has a complex pathophysiology culminating in an inflammatory response that can lead to irreversible intestinal injury [110,111]. While the pathophysiology of NEC is incompletely understood, it has been suggested that there is an association between biomarkers of intrauterine OS events and NEC [112]. As indicated, OS is difficult to measure in vivo, such that correlational biomarkers are used to evaluate host susceptibility to OS by measuring oxidant-induced changes in proteins, lipids, and DNA [113]. Cord blood concentration and intestinal levels of biomarkers of OS have been demonstrated in infants with NEC, establishing an association between perinatal OS and NEC [112,113,114]. Preliminary evidence would suggest a role for postnatal IH as a contributing risk factor in NEC pathophysiology [115,116].

## 6. Therapeutic Approaches

**Figure 2:**
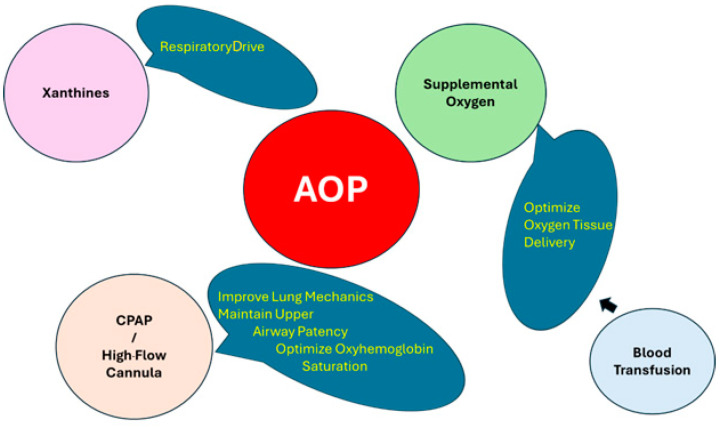
Treatment strategies for apnea of prematurity include a multi-prong approach including xanthines, supplemental oxygen, blood transfusion, and noninvasive ventilation.

Management strategies for AOP with IH events focus primarily on prevention. Desaturation events are a consequence of immature respiratory control superimposed upon an immature respiratory system [71]. There is no consensus on when to initiate therapy for AOP. Generally, treatment is indicated when episodes are recurrent and are associated with bradycardia or desaturation [58]. Two mainstays of management are optimizing oxygenation and enhancing respiratory control (Figure 2).

### 6.1. Optimizing Oxygenation

The optimal target oxygen saturation for the preterm infant has not been definitively defined [58,117,118]. Higher oxygen levels may increase survival but increase morbidities, including ROP and BPD. Conversely, lower oxygen levels might increase the likelihood of NEC and death but reduce the risk of problems caused by oxygen toxicity [118]. The benefits of keeping baseline oxygenation in preterm infants at levels of SpO2 90–95% outweigh the risks when restricting the upper limits of SpO2 to <89% [119,120]. Low baseline SpO2 increases the risk of intermittent hypoxemia in the preterm neonate [121], further aggravating the risk of augmenting the maladaptive effects and oxidative stress associated with such occurrence.

The challenges of maintaining SpO2 in a target range with manual titration of inspired oxygen have been described [122]. New devices with automated control of oxygen titration have been described to reduce the incidence of prolonged periods of hypoxemia and hyperoxemia [123]. A recent randomized crossover study, however, demonstrated that automated control with apnea-triggered FiO2 boost resulted in a reduction in post-apnea hypoxemia, but was followed by a greater period of SpO2 overshoot [124].

### 6.2. Methylxanthine Therapy

Methylxanthines have been the mainstay of treatment for AOP for over 40 years. They act both centrally and peripherally to stimulate respiration and reduce the frequency of apneic episodes. They primarily work through antagonism of adenosine receptors, blocking both excitatory A2A receptors on GABAergic neurons and inhibitory A1 receptors [125,126,127]. They further improve respiratory dynamics by activating medullary respiratory centers, increasing CO_2_ sensitivity, inducing bronchodilation, and enhancing diaphragmatic contraction. Caffeine administration can also modulate inflammation by inhibiting cyclic nucleotide phosphodiesterase and increasing the concentration of cAMP [125,128]. Early studies identified the effectiveness of aminophylline and theophylline in treating AOP, but caffeine has become the preferred treatment given its fewer side effects, wider therapeutic index, and longer half-life that allows for once-daily dosing [129,130].

Despite the widespread use of caffeine, there is variation in clinical practice regarding the optimal timing of initiation, dose, and duration of treatment. In the Caffeine for Apnea of Prematurity (CAP) Trial, the benefits of caffeine were the greatest when therapy was initiated within 3 days after birth [131]. These benefits include reductions in the incidence of BPD, decreased severity of ROP, and improved long-term neurodevelopment [132]. Early use of caffeine has become frequent in the treatment of AOP in the extremely preterm infant and may help dampen this trajectory of increasing frequency of AOP after the first week of life and perhaps limit the magnitude of oxidative stress associated with it [133].

There is also clinical variability in the duration of caffeine treatment. Many clinicians look at corrected gestational age and timing of clinically documented episodes when deciding to discontinue treatment. Therapy is often discontinued around 34–35 weeks post-menstrual age (PMA) at which time the frequency of apneic events typically declines. However, it is well known that nursing documentation of events grossly underestimates the number of cardiorespiratory events [3]. Furthermore, despite a decrease in clinically apparent events after 34 weeks PMA, it has been shown that neonates born <28 weeks still have a significant amount of IH until 43–44 weeks corrected gestational age (CGA). In a multicenter, prospective, randomized trial, Rhein et al. demonstrated that continued caffeine therapy decreased the amount of IH at 35 and 36 weeks CGA by almost 50% [130]. A subsequent study by the same group demonstrated similar findings at 36–38 weeks using a higher dose of caffeine [134]. Another study was conducted to compare the incidence of recurrent AOP after discontinuation of caffeine in a short-course therapy group and a long-course group. There were fewer episodes of hypoxia in the long-course group but an increase in adverse effects of the medication [135]. Because of the immaturity of the metabolic capacity of the hepatic enzyme system during the first weeks of life, most caffeine is excreted unchanged by the kidneys [136]. This observation indicates that newborn infants have a deficiency in their caffeine metabolizing capacity until several months of age when liver enzymes increase to adult levels in their ability to produce demethylated metabolites and caffeine clearance. As a result, the half-life of caffeine decreases from 120 h to 60 h within the first 8 postnatal weeks [137]. A recent review summarized the positive effects when caffeine is administered to the hypoxic rodent, but also the negative effects on the rodent model when caffeine is administered without exogenous oxidative stress. Thus, critical consideration needs to be given to continuing caffeine treatment beyond the recommended corrected gestational age in the preterm infant [138]. Further studies are needed to determine the optimal dosage and ideal time for discontinuation of caffeine therapy.

### 6.3. Noninvasive Respiratory Support

Nasal continuous positive pressure (CPAP) has been a major therapeutic intervention to treat AOP. It works by splinting open the upper airway to improve patency and increase functional residual lung capacity [139]. More recently, heated humidified high-flow nasal cannula (HHFNC) therapy has been used interchangeably with CPAP [140]. Small studies have found that it delivers similar distending pressures with no differences in the rate of apneic events while providing a more comfortable interface for the neonate [59]. Nasal intermittent positive pressure ventilation (NIPPV) may also be effective in the treatment of AOP and has been compared to CPAP with mixed results [141,142]. However, larger studies are needed to delineate true differences in the effectiveness of these various noninvasive respiratory support strategies in reducing AOP and IH.

In addition to caffeine and noninvasive respiratory support, a variety of other pharmacologic and non-pharmacologic strategies have been proposed in the management of AOP, but because of concerns for side effects, these treatments are not widely employed [58].

### 6.4. Red Blood Cell Transfusions

Anemia may exacerbate apnea by reducing the oxygen-carrying capacity of blood, decreasing oxygen delivery to the brain [58]. A retrospective study utilizing waveform analysis demonstrated that a short-term lower hematocrit level was associated with an increased frequency of AOP [143]. Another study demonstrated a decrease in IH following packed red blood cell transfusion [144]. A possible side effect of red cell transfusion, however, includes the increased risk of NEC [145] and ROP [146,147]. Lust et al. hypothesized that the increase in adult hemoglobin following transfusion could result in a shift of the oxygen disassociation curve resulting in increased retinal tissue oxygen delivery at a time when it is susceptible to hyperoxia [147].

### 6.5. Doxapram

Doxapram, a central respiratory stimulant with a brief duration of action, has been used in the management of AOP especially in those neonates resistant to methylxanthine therapy, but because of adverse gastrointestinal and CNS symptoms, it is rarely used today [148]. A recent study demonstrated that in children ages 5–6 years born prior to 32 weeks gestation, doxapram treatment for AOP was not associated with neurodevelopmental disabilities [149]. There is currently a multicenter randomized controlled trial to examine the efficacy and safety of doxapram in the treatment of AOP in preterm infants [150].

### 6.6. Acid Suppression Medication

Ranitidine and other pharmacologic treatments to reduce gastric acids have been tried, yet the relationship between acid reflux and AOP has not been established [151]. There has been a described relationship between H2 receptor antagonists and necrotizing enterocolitis and late-onset sepsis [152].

### 6.7. Therapeutic Handling

Prone positioning can stabilize the chest wall and allow for improved ventilation of the lower lobes; however, there is no evidence that it reduces the frequency of AOP [153]. Tactile stimulation including skin-to-skin (kangaroo) care has also been shown to decrease AOP [154,155]. A multicenter neonatal manikin study was undertaken to evaluate tactile stimulation for AOP that demonstrated a large heterogeneity and recommended further prospective studies to provide guidelines for the most appropriate approach [156]. These approaches follow a long set of antecedent trials examining the impact of olfactory, vibratory, kinesthetic, and stochastic resonance methodologies aimed at reducing the probabilistic occurrence of apnea in neonates [157,158,159,160].

## 7. Conclusions

As our understanding of oxidative stress, its damage to cell membranes, and its role in epigenetic modifications evolves, so does our knowledge of its potential impact on all organ systems. This is of particular concern regarding the preterm neonate, who is at a high risk for oxidative damage given the imbalance of pro-oxidative stressors and immature antioxidant systems in combination with the rapid development that occurs postnatally. Apnea of prematurity is ubiquitous in those born <28 weeks gestation, and the intermittent hypoxia that ensues is a large contributor to the oxidative threat experienced by these high-risk neonates. Future studies examining the role of oxidative stress in the overall morbidity catalog of premature infants and the contribution of AOP and its surrogate effector IH are needed.

## Figures and Tables

**Table 1:** Potential risk factors implicated in the pathogenesis of apnea of prematurity.

Central Factors	Peripheral Factors
Reduced central chemosensitivity	Variable carotid body and peripheral chemosensitivity responses
Augmented hypoxic ventilatory depression	Laryngeal chemoreflex
Upregulated inhibitory neural networks	Hering–Breuer and diving reflexes
Immature vasomotor regulatory function	Laryngeal chemoreflex
Neural connectome immaturity	Autonomic dysmaturity facilitating bradycardia
Sensory integration deficits	Reduced diaphragmatic endurance
	Increased airway collapsibility
	Reduced functional residual capacity of lungs and diminished oxygen reserve
